# Effectiveness of electrical stimulation with conservative treatment for lower urinary tract symptoms in Parkinson's disease: A three-armed randomized controlled trial protocol

**DOI:** 10.1016/j.conctc.2025.101480

**Published:** 2025-03-29

**Authors:** Dorien Bennink, Rob A. de Bie, Henk W. Elzevier, Dagmar H. Hepp, Gommert A. van Koeveringe, Anton A. van der Plas, Hein Putter, Maxime T.M. Kummeling, Heidi F.A. Moossdorff-Steinhauser

**Affiliations:** a Department of Urology, Leiden University Medical Center, Leiden, the Netherlands; b Department of Epidemiology, Caphri – Care and Public Health Research Institute, Maastricht University, Maastricht, the Netherlands; c Department of Neurology, Leiden University Medical Center, Leiden, the Netherlands; d Department of Urology, Research Institute for Mental Health and Neuroscience, Maastricht University and Maastricht University Medical Center+ (MUMC+), Maastricht, the Netherlands; e Department of Neurology, Alrijne Hospital, Leiderdorp, the Netherlands; f Department of Biomedical Data Sciences, Leiden University Medical Center, Leiden, the Netherlands

## Abstract

**Background:**

Despite the high prevalence of lower urinary tract symptoms (LUTS) in patients with Parkinson's disease (PD)—ranging from 27 % to 85 % including symptoms such as urinary urgency,- incontinence, frequency, and nocturia—evidence-based treatment options remain limited. Conservative treatments, such as bladder training, pelvic floor muscle exercises (PFME) with biofeedback and electrical stimulation, have been shown safe and effective in the general population, with minimal side effects. However, their efficacy specifically in PD patients remains unclear. Therefore this study aims to evaluate the effect of electrical stimulation with conservative treatment for LUTS in PD patients.

**Methods and analysis:**

This randomized controlled trial includes three study arms. All three groups will receive conservative treatment in combination with different electrical stimulation parameters, small- and broad pulse duration and sham electrical stimulation. In total 150 PD patients with self-reported LUTS who are able to attend a pelvic physical therapy practice independently and complete online questionnaires will be enrolled. The primary outcome is the difference in international prostate symptom score (IPSS), with a range of 0–35.

A minimal important difference of 4.2 between baseline and 12 weeks of treatment will be statistical significant (p˂0.05). Secondary outcome include questionnaires evaluating bladder dysfunction, burden, and quality of life and will be collected at baseline, 12 weeks and 24 weeks and at one year. Additionally pelvic floor muscle function will be assed at baseline and after 12 weeks.

All participants receive eight sessions along with their assigned electrical stimulation treatment and conservative treatment.

## 1 Introduction

Parkinson's disease (PD) is one of the fastest growing neurodegenerative disorders. It is estimated that PD affects 1 % of the population over the age of 60 years [1,2]. PD comprises a variety of motor symptoms and a wide range of non-motor symptoms (NMS), including several autonomic dysfunctions, which accumulate as the disease progresses. Lower urinary tract symptoms (LUTS) are reported in 27–85 % of PD patients, can occur early in the disease, 50 % at onset, and have a significant impact on the quality of life (QoL) of patients [[3], [4], [5], [6], [7], [8], [9]].

LUTS are divided into three groups, storage, voiding and postmicturition symptoms [10]. Both central cortical areas and peripheral nerves in the pelvis regulate the urinary bladder and bladder outlet [11]. Although the exact pathophysiology remains unclear, LUTS in PD are believed to have a multifactorial origin, reflecting the widespread involvement of multiple areas within the nervous system [12].

The majority of PD patients experience storage symptoms (formerly known as irritative symptoms) such as nocturia, the need to urinate more than once per night (57–86 %), urinary frequency (32–71 %), urinary urgency (UU) (32–68 %) and urgency urinary incontinence (UUI) (21–40 %) [4,8,13,14]. Symptom prevalence is significantly higher in PD patients than in the general population [4,15,16]. LUTS increases with the progression of PD and may lead to early admission into care and an increase of health related cost [12]. Additionally, LUTS exerts a profound influence on health-related QoL even in patients who have only recently been diagnosed [17,18]. In general, LUTS and the associated fear of urinary incontinence have a limiting effect on daily activities and may act as a barrier to exercise and sports participation [19]. A significant association has been observed between these symptoms and an increased risk of falls [20].

According to clinical guidelines, treatment of LUTS in general population consists of conservative therapy and medication such as antimuscarinics and β-3-adrenergic receptor-agonist [12,21]. Conservative treatment may hold component such as behavioral advice, bladder training, pelvic floor muscle exercises (PFME) with or without biofeedback and electrical stimulation (ES) [22,23]. Antimuscarinic agents have proven to be effective in alleviating LUTS in general population. However in PD patients these drugs are less effective, less well tolerated, and have more serious side effects like constipation, cognitive decline and xerostomia [[24], [25], [26]]. Current guidelines for the management and treatment of LUTS in PD scarcely mention conservative therapy as an option, due to a lack of powered randomized controlled trials [20,27]. Some smaller studies show promising results such as significant improvements in voided volume and reduction in number of voids, for bladder training and PFME with biofeedback in patients with PD [28,29]. Biofeedback can be provided with electromyography (EMG), using an intra vaginal- or anal probe. EMG measures electrical activity of the pelvic floor muscles (PFM) and provides visual feedback to help patients learn to contract these muscles effectively and suppress UU [29]. The major advantage of this form of therapy is the absence of side effects.

Neuromodulation can also be applied to reduce UU and UUI. The mechanism of neuromodulation, to reduce UU, is hypothesized to involve the reflex inhibition of detrusor contractions through activation of afferent fibers of the pudendal nerve and sacral cord. **Afferent fibers can be stimulated via the pudendal nerve**, **sacral nerve roots (S3 and S4) or other peripheral nerves corresponding to these roots** [23]. Neuromodulation can be applied in several ways, including surface electrodes, like transcutaneous tibial nerve stimulation (TTNS), needle electrodes such as percutaneous tibial nerve stimulation (PTNS) and implanted wire electrodes, i.e. sacral neuromodulation (SNM).

In a small number of studies, PTNS and TTNS have demonstrated potential benefits in reducing UU in PD, however there is uncertainty regarding long-term effects [[30], [31], [32]]. SNM shows promising results, but research on its use for PD patients is still limited and the number of patients studies is small [33].

Add-on ES, to conservative therapy with the same intra vaginal- or anal probe, to reduce UU could be of interest and might be beneficial in PD. The treatment is well-tolerated by patients in the general population, has a short duration and is not expected to cause serious adverse effects [34].

In general, when using ES for neuromodulation, there is a lack of knowledge regarding the optimal parameters for its use [23]. Frequency is typically set at a relatively low rate, below 20 Hz, while the pulse duration varies across a broad spectrum, ranging from 100 to 1000 μs [23,35,36].

Given this knowledge gap, the primary objective of this study is to evaluate the effectiveness of ES, using two distinct electrical stimulation -one with a short pulse duration and one with a broad pulse duration-compared to sham stimulation together with PFME and biofeedback in reducing LUTS in PD.

## 2 Methods

### 2.1 Trial design

This is a monocenter three-armed randomized controlled trial (RCT). All participants receive bladder training and PFME with biofeedback. In three groups the efficacy of two different active parameters of ES are compared with sham ES.

The three study arms are:

Group 1: ES with phase duration 200μs, frequency 20hz, 20 min continuous mode, active electrodes on the ventral side of the probe;

Group 2: ES with phase duration 1000μs, frequency 8hz, 20 min continues mode, active electrodes on the ventral side of the probe;

Group 3: ES with phase duration 200μs, frequency 100hz, 20 min intermittent mode: with 2 s of active ES and 20 s no stimulation, with active electrodes on the dorsal side of the probe, sham ES. (control group).

### 2.2 Participants: eligibility and recruitment

Inclusion criteria are: Parkinson's disease, ≥18 years of age, self-reported LUTS, stable Parkinson's and/or bladder medication for at least three months, including alpha-blocker medications, sufficient understanding of the Dutch language, able and willing to independently read and fill in online questionnaires, able to independently visit a pelvic physical therapy practice.

Exclusion criteria are: other neurological diseases, surgery in the pelvic region in the last year, cancer or cancer treatment in the pelvic region, pregnancy, current urinary tract infection, pure stress urinary incontinence without urinary-urgency, -frequency, nocturia, Botulinum toxin treatment, PTNS or pelvic physical therapy in the last year, sacral neuromodulator, pacemaker and implantable cardioverter defibrillator (ICD), Deep Brain stimulation (DBS).

Participants are recruited through advertisement on the website of the Dutch Parkinson association and the Dutch brain association, with flyers in physical therapy practices, and by presentations for small local patient groups. Potential participants can register for the study, without the intervention of a healthcare provider.

Neurologists, urologists, and the PD nurse at LUMC, Maastricht University Medical Center (MUMC+), and Alrijne hospital (Leiden) inform potential participants about the study and provide informational leaflets.

### 2.3 Outcome measures

#### 2.3.1 Questionnaires

Questionnaires (Table 1) will be sent to the participant online at T0, T1 (12 weeks), T2 (24 weeks), and T3 (one year).

• The International Prostate Symptom Score (IPSS), validated for use in both men and women [37,38]. This questionnaire is a standard tool for assessing Lower Urinary Tract Symptoms (LUTS) in the general population. Although it is not validated in PD, it is the only questionnaire that has been referenced in existing research related to PD, providing some relevant context for this population [3,6,39]. It contains 7 items on storage of urine and micturition and is scored on a 6 point scale. An 8th, separate question assesses QoL and will not be added to the score. The total score on questions 1–7 lies between: 0–35 points. The higher the score, the more severe the symptoms in which a score between 0 and 7 points is interpreted as mild symptoms, a score between 8 and 19 points as moderate symptoms and a score between 20 and 35 points as severe symptoms [40].
• The International Consultation on Incontinence Questionnaire-Urinary Incontinence Short form (ICIQ-UI SF), evaluating four questions about frequency, urinary incontinence and amount of leakage and overall impact of urinary incontinence. The score ranges from 0 to 21 points with greater values indicating increased symptom severity, level of evidence is grade A [41].
• The International Consultation on Incontinence Questionnaire-Lower Urinary Tract Symptoms quality of Life (ICIQ-LUTSqol). It provides a measure to assess the impact of urinary incontinence on QoL with particular reference to social effects level. The score ranges from 19 to 76 points with higher values indicating increased impact on QoL.The amount of bother is measured on a 0–10 point Likert scale. They are not incorporated in the overall score but indicate impact of individual symptoms for the patient, level of evidence grade A [42].
• The International Consultation on Incontinence Questionnaire-Overactive Bladder (ICIQ-OAB), for questions evaluating UU with and without urinary incontinence, associated with frequency and nocturia. The score ranges from 0 to 16 points with higher values indicating increased symptom severity. The ICIQ-OAB is validated in Dutch, level of evidence grade A [43,44].

**Table 1:** Summary of questionnaires and pelvic floor muscle assessment.

Assessment schedule	T0 baseline	T1 12 weeks	T2 24 weeks	T3 1 year
Baseline characteristics	X			
IPSS	X	X	X	X
ICIQ-UI SF	X	X	X	X
ICIQ-LUTSqol	X	X	X	X
ICIQ-OAB	X	X	X	X
GPE		X		
24 h bladder diary	X	X		
Pelvic floor muscle assessment	X	X		

IPSS=International Prostate Symptom Score, ICIQ-UI SF=International consultation on Incontinence- Urinary Incontinence Short Form, ICIQ-LUTSqol = International Consultation on Incontinence Questionnaire-Lower Urinary Tract Symptoms quality of life, ICIQ-OAB=International Consultation on Incontinence Questionnaire Overactive Bladder, GPE = Global Perceived Effect.

Outcome measures (table 1) assessed at inclusion (T0) and at 12 weeks (T1).

• The 24 h bladder diary [45], records time of:
  o micturition: bladder volume (ml) and frequency,
  o rate of UU
  o leakage of urine
  o number of drinks: every time how much (amount in ml)
• The PFM-assessment: according to International Continence society (ICS) [46].
  o PFM palpation: tone at rest, maximum voluntary contractions (MVC), endurance contractions (EC), co-reflex-contractions
  o EMG assessment: tone at rest, 10 MVC's, 3 EC.

Outcome measure (table 1) assessed at 12 weeks (T1).

• Global Perceived Effect (GPE), to quantify the patient's perceived improvement or deterioration over time, (T1), measured on a 7 point scale [47].

### 2.4 Pelvic floor muscle assessment

Participants will be asked to perform a PFM contraction without contraction of surrounding (gluteal, abdominal) muscles. Initially, no instructions will be given on how to perform a PFM contraction. In case of absence, no proper contraction or co-contraction, the pelvic physical therapist (PPT) will provide clear instructions on how to perform a correct PFM contraction.

Assessment of the PFM, per vaginum/anum, consists of.

• Muscle tone of m.levator ani and external anal sphincter.
• PFM strength: three MVC's and three EC's
• Repeatability of 10 fast contractions
• Direction of pelvic floor movement: elevation, no change, descent
• Relaxation post contractions: yes; partial or delayed; no, absent
• Co-ordination: PFM contraction before a cough: present, absent or paradoxical contraction
• Co-contractions: yes: abdominal, gluteal or no: absent

According to the terminology for PFM assessment by Frawley et all [46].

EMG assessment will be performed with the Multi Array Probe Leiden (MAPLe). The MAPLe® is a monopolar cylindrical-shaped validated probe (length 65 mm, diameter 15 mm) with 24 electrodes. It is capable of registering EMG activity nearest to the individual muscles of the PFM on the four different sides and depths [26].

The probe will be placed intra-vaginally or intra-anally with a reference electrode on the anterior superior iliac spine. Participants will be asked to perform three consecutive tasks.

1. One minute rest to register baseline muscle activity. Participants will be instructed not to speak during the measurement and breathe calmly.
2. Ten MVC's. Participants will be instructed to perform a controlled MVC the PFM, without co-contractions (mean and peak amplitude will be measured).
3. Three EC's. The participants will be instructed to contract the PFM at such level that they can hold for 30 s (mean and peak amplitude will be measured).

### 2.5 Routing and study procedures

Interested PD patients are encouraged to contact the principal investigator (PI) by phone or email. The PI will provide the patient with detailed information, both verbally and in writing. The patient will have the opportunity to ask questions and receive additional information throughout the enrolment process.

**Fig. 1:**
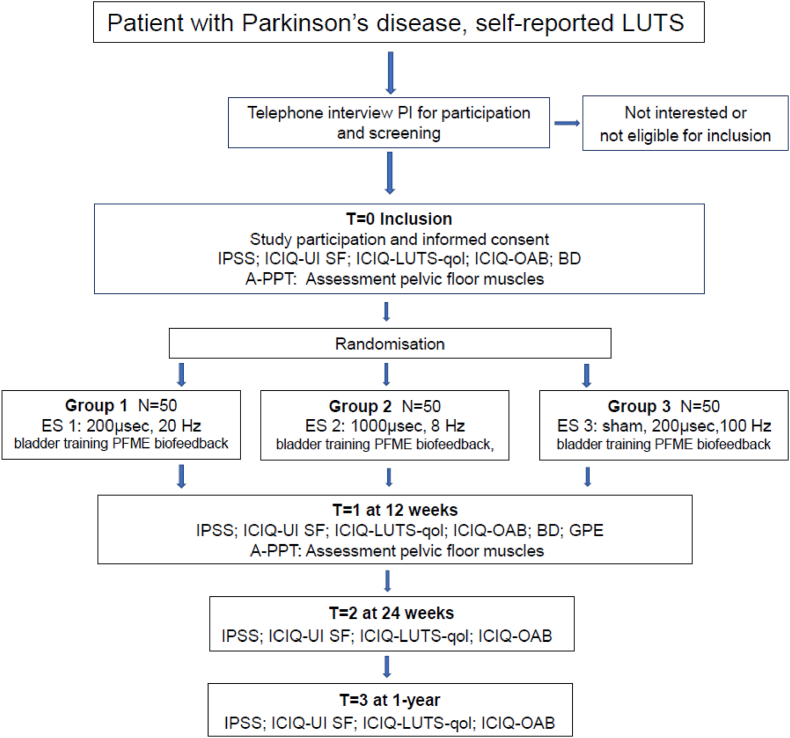
Study design flow diagram LUTS: lower urinary tract symptoms; IPSS: international Prostate symptom score; ICIQ-UI SF: International Consultation on Incontinence Questionnaire urinary incontinence short form; ICIQ-LUTS-qol: International Consultation on Incontinence Questionnaire Lower urinary tract symptoms quality of live; ICIQ-OAB: International Consultation on Incontinence Questionnaire Overactive Bladder; BD: bladder diary; A-PPT: Assessment Pelvic Physical Therapist; ES: electrical stimulation; PFME: pelvic floor muscle exercise.

The PI verifies eligibility criteria are met (Fig. 1). If the participant is eligible, the research information sheet, two informed consent forms, a reimbursement form, and a return envelope are sent to the interested PD patient. Once both signed informed consent forms have been returned, the patient will be included (Fig. 1). The PI will sign both informed consent forms, return one to the participant, and securely store the other in a locked cabinet. All included participants will receive a reimbursement of €50. Finally, demographic data and participant characteristics will be registered.

Data will be stored and managed in a secured online database system Castor EDC (www.castoredc.com).

At baseline (T0) and post-treatment T1) the participant will complete online questionnaires and undergo a PFM assessment (Table 1).

The PFM assessments will be carried out by one of the six assessment(A)-PPT at six sites across The Netherlands. These six A-PPTs are experienced in scientific research and have a Master's degree in Pelvic Physical Therapy [48].

The pelvic physical therapy intervention, largely based on usual care Pelvic Physical Therapy, will be provided by a protocol trained local PPT (L-PPT) close to the participants' home address. The PI has a list of protocol trained L-PPT's that can be contacted. In exceptional cases the A-PPT and the L-PPT is the same person.

The PI oversees the organization of the project and ensures that the A-PPT and L-PPT receive the required information.

After inclusion the A-PPT will make two appointments (T0 and T1) with the participant, with a 12 weeks interval.

The PFM assessment will consist of a standardized protocol: digital palpation per vaginum/rectum and an EMG assessment of the PFM [46]. The A-PPT is responsible for entering data of the PFM assessment and bladder diary in the Castor database.

After baseline assessment, participants will be randomly assigned in blocks with a computer generated program Castor EDC (www.castoredc.com). Participants will be stratified by gender and allocated (1:1:1) into one of the three study arms (Block size vary between 6,9,12). The randomization codes are software generated before start of the intervention. The PI has no influence on the randomization process.

Communication between PI, A-PPT's and L-PPT's will be via a secure app and email.

The two appointments with the A-PPT are funded by the grant, while the treatment is covered by basic healthcare insurance.

The personal probe used for EMG assessment and ES will be provided free of charge.

Participants may withdraw from the study at any time for any reason, without consequences. The research team may decide to withdraw a subject from the study for urgent medical reasons.

### 2.6 Intervention and blinding

The intervention consists of eight protocol-based treatment sessions of 30 min each over a period of 10–12 week. The protocol consists, for all participants, of bladder training [22], biofeedback assisted PFME, MVC's and endurance contractions according to the (dys)function of the PFM and the ability of the individual participant. It also includes relaxation and coordination exercises of the PFM combined with abdominal breathing. Visual feedback of the EMG signals with verbal instruction and reinforcement will be used to teach participants how to control the PFM and how to use the PFM for urge suppression techniques.

For urge suppression, the mean of the peak values of the 10 MVCs obtained during the EMG assessment is used. Half of this mean is displayed as a reference red, top line on the screen, providing visual feedback to both the participant and the therapist. Another red, bottom line, representing the mean baseline muscle activity from the EMG assessment, is used for relaxation. During the intervention, the red line will be adjusted in case of increasing PFM activity in MVC's and decreasing activity in case of more relaxation.

ES will be applied for 20 min, in six therapy sessions.

Participants will have to exercise (approximately 10 min per day) at home in between sessions and learn to integrate the techniques in case of UU and into their daily activities.

Participants are blinded for the treatment, but blinding is impossible for the A- and L-PPT's. Data analysis is conducted by both researcher and statistician, with the statistician being fully blinded.

### 2.7 Patient involvement

Two patient-researchers, members of the Dutch Parkinson Patient Association were consulted in several online meetings to discuss the project idea and research protocol. In particular, their input was sought specifically on the comprehensibility of the patient information, the questionnaires, the Bladder Behavior Information Form, and the PFME.

In addition, feedback from the treatment of 15 patients, according to the treatment protocol, in earlier stages was also consequently incorporated. The eight treatment sessions and electrical stimulation were both well received and tolerated.

## 3 Study outcomes

### 3.1 Primary outcome

The effect of ES combined with PFME and biofeedback will be evaluated based on LUTS symptoms and the change in IPSS score from baseline (T0) at 12 weeks (T1) across the 3 study arms.

Secondary outcomes involve of incontinence (ICIQ-UI SF; quality of life (ICIQ-LUTSqol and ICIQ-OAB), 24 bladder diary), PFM function, including palpation per vaginum/anum and EMG assessment (Table 1).

### 3.2 Sample size calculation and statistical analysis

Given the limited available evidence, the sample size calculation is based on the minimal clinically important difference (MID) of the IPSS-score on LUTS, as estimated in previous studies. Ruffion et al. [42] estimated a MID of 3 points with a standard deviation of 4 in men, while Blanker et al. [43] reported an MID of 5.2 with a standard deviation of 6.7 in men. However, to the best of our current knowledge, there are no estimated MIDs available for women or for conservative therapy.

In PD the IPSS is often used and appears to show a higher score than healthy controls [6,49]. In a recent study, including PD patients with self-reported LUTS the mean score was 13.5 [31].

To study the effect of ES, combined with PFME and biofeedback, for each of the two interventions compared to sham ES and given the expected effect of PFME, a MID of 4.2 will be used and a SD of 6.7 per group is expected.

In our study we will study the effectiveness of ES making two comparisons:

Comparison 1: group 1 (small phase duration) versus group 3 (sham ES) and.

Comparison 2: group 2 (broad phase duration) versus group 3 (sham ES).

In a secondary analysis we also want to compare group 1 against group 2.

Given this 3-arm design, Dunnett's closed testing procedure is used, therewith correcting for multiple testing [50]. This entails that first looking at the test statistics belonging to the two contrasts of main interest [1] vs [2,3] vs [3]. The maximum test statistic of these two will be tested against the critical value belonging to Dunnett's procedure. If this overall/gatekeeping test is rejected, the remaining two contrasts can be tested against regular critical values matching two-sided alpha = 0.05. If the maximum test statistic does not lead to rejection, this implies none of the contrasts are rejected.

The overall power of detecting a MID of 4.2 for at least one of the main comparisons [1] vs [2,3] vs [3] was assessed using Monte Carlo simulation.

Group sizes of 40, 40 and 40, achieved 84 % power for detecting any difference (so either [1] vs [3] or [2] vs [3] or both) and 66 % power for detecting both differences [1] vs [2,3] vs [3] assuming an alternative hypothesis of a mean difference of 4.2 between [1] vs [3] and same difference between [2] vs [3].

Given that the study will be considered successful if at least one effective intervention is found, this achievable power was considered satisfactory.

Regarding attrition, we estimate a loss-to-follow-up of 20 %. Therefore, with 40 participants per group needed in the analysis, 150 participants will be recruited (50 per group).

The primary analysis will use linear mixed models, which accounts for missingness due to study drop-out, under the assumption of missing at random (MAR). Other missing data will be imputed using multiple imputation for intention-to-treat analysis if more than 5 % is missing, with the number of imputations based on the percentage of incomplete patients and relevant variables.

### 3.3 Data management and data protection

After inclusion, all data will be stored in a secure, online database system (www.castoredc.com). Every participant is given a unique identifying trial number. The key to the sequential participant code (a combination of country, institute code, study code and sequential participant number) to the participant data is safeguarded by LUMC.

The handling of the data complies with the EU General Data Protection Regulation and the Dutch Act on Implementation of the General Data Protection Regulation. (in Dutch: Uitvoeringswet AVG, UAVG).

The project leader and PI will have access to the complete database, as well as a monitor of the internal monitor pool of the LUMC and the “Health and Youth Care Inspectorate”.

LUMC data management guidelines will be followed and we strive to make our data FAIR (Findable, Accessible, Interoperable and Reusable) as described in a data management plan.

The data sets will be accessible in agreement with the given informed consents and other agreements in place. Data will be made (publicly) accessible following the funder's guidelines.

The data collected for the trial will consist of baseline characteristics, questionnaires, PFM assessment and EMG assessment (Table 1).

### 3.4 Data safety monitoring

Monitoring will be executed by (internal) monitors of the LUMC according to the monitor plan. Due to the “low risk trial” character of the study no Data Safety Monitoring Board, no formal stopping rules for the trial and no interim analyses are needed.

## 4 Harms

This study is considered to be a low risk trial. The risk of occurrence of side-effects is low [[51], [52], [53]].

Adverse events (AE's) are defined as any undesirable experience occurring to a subject during the study, related to EMG measurement and treatment (biofeedback and ES) with an anal or vaginal probe. Only study related AE's reported spontaneously by the subject or observed by the investigator or his staff will be recorded.

Due to the intervention, hardly any serious AE's (SAEs) are to be expected. Only study related (S)AE's, suspected unexpected serious adverse, will be reported. This means all (S)AE's related to participation in this study protocol, meaning from PFM assessment, EMG assessment, biofeedback and ES treatment. All other (S)AE's will not be reported, as no participant benefit is expected from this.

The sponsor will report the SAE's through the web portal *ToetsingOnline* to the accredited METC that approved the protocol.

## 5 Ethical approval and dissemination

The study protocol has been approved by the Medical Ethics Review Committee of the Leiden University Medical Center (P22.009).

Dissemination meetings are arranged with the research team (authors) and the A-PPT's.

## 6 Discussion

To our knowledge, this is the first RCT evaluating conservative therapy for LUTS in PD. Despite the significant impact of LUTS on QoL, evidence-based interventions for LUTS in PD are limited and needed.

This RCT examines the efficacy of intra-vaginal and intra-anal ES with varying parameters, to reduce UU, frequency and UUI, in conjunction with bladder training and PFME with biofeedback. If this intervention proves effective, it could significantly improve the QoL of PD patients experiencing LUTS. Furthermore, this standard care treatment is readily accessible for all PD patients.

The selection of study parameters is only moderately supported by evidence. A wide range of parameters are used in scientific research. The frequencies employed typically fall within a narrow range of 2–20 Hz. The 20 Hz frequency combined with a 200 μs pulse duration is commonly used in PTNS/TTNS therapies. An 8 Hz frequency with a phase duration of 1000 μs has been used in a previous study [52]. Both 200 μs and 1000 μs pulse durations are at the extremes of the spectrum of phase durations that are typically used. The Sham ES uses a frequency of 100 Hz, which is not expected to have an effect on the UU. Additionally, stimulation is applied to the dorsal side of the pelvic floor to minimize any potential impact.

During the design of this study, various factors were considered, including time investment and burden for participants, evidence supporting PPT interventions, the feasibility of the treatment protocol by PPT's, and the study methodology.

Representatives from the patients' association, as well as medical specialists, PPT's, and nurses, actively participated in the development of the treatment protocol.

This study has limitations. With six A-PPT's across The Netherlands, the travel distance to the A-PPT's remains too great for some potential participants. Furthermore, there is a discrepancy between the perceived LUTS and the measurable severity of these symptoms. To the best of our knowledge, there is no clear cutoff point in LUTS questionnaires, which affects the inclusion of participants and may introduce bias.

Should this study demonstrate that ES combined with PFME and biofeedback is effective, a new conservative treatment option for LUTS in PD would be available that is also easily accessible for patients.

## CRediT authorship contribution statement

**Dorien Bennink:** Writing – review & editing, Writing – original draft, Project administration, Methodology, Funding acquisition, Conceptualization. **Rob A. de Bie:** Writing – review & editing, Methodology, Funding acquisition, Conceptualization. **Henk W. Elzevier:** Writing – review & editing, Supervision, Methodology, Funding acquisition, Conceptualization. **Dagmar H. Hepp:** Writing – review & editing, Methodology, Conceptualization. **Gommert A. van Koeveringe:** Writing – review & editing, Methodology, Funding acquisition, Conceptualization. **Anton A. van der Plas:** Writing – review & editing, Funding acquisition, Conceptualization. **Hein Putter:** Writing – review & editing, Methodology, Funding acquisition. **Maxime T.M. Kummeling:** Writing – review & editing, Funding acquisition, Conceptualization. **Heidi F.A. Moossdorff-Steinhauser:** Writing – review & editing, Writing – original draft, Methodology, Funding acquisition, Conceptualization.

## Trial registration

Registered at clinicaltrials.gov (Trial registration: NCT05814614 Date of registration:/First Posted: 18-04-2023.

## Trial status

Recruitment started at September 2023, the estimated end date of the last recruitment is august/medio 2025.

## Conflicts of interest and source of funding

None of the authors have any conflicts of interest to declare. This study is funded through a grant by the “Koninklijk Nederlands Genootschap voor Fysiotherapie” (KNGF) and the “Hersenstichting” in 2023.

## Declaration of competing interest

The authors declare that they have no known competing financial interests or personal relationships that could have appeared to influence the work reported in this paper.
